# 3D LGE imaging of the LV short axis stack using spiral readouts at 3T

**DOI:** 10.1186/1532-429X-16-S1-P8

**Published:** 2014-01-16

**Authors:** Iain Pierce, Jennifer Keegan, Peter Drivas, David Firmin

**Affiliations:** 1 NHLI, Imperial College London, London, UK; 2 Cardiovascular BRU, Royal Brompton Hospital, London, UK

## Background

Clinical assessment of the viability of myocardium is commonly performed using 2D breath-hold late gadolinium enhancement (LGE) imaging with Cartesian k-space coverage and data acquisition windows (AWs) ranging from 140-200 ms. More efficient k-space coverage using spiral trajectories could allow improved spatial resolution and reduced AWs without greatly extending acquisition durations. To this end, we have developed a navigator-gated spiral 3D LGE sequence and present the results in 10 patients attending for clinical 2D LGE imaging on a 3T Siemens Skyra scanner.

## Methods

The navigator-gated 3D sequence consisted of a stack of spirals (acquired: 8 kz at 1.4 × 1.4 × 10 mm, reconstructed: 16 slices at 0.7 × 0.7 × 5 mm). Each spiral readout was 10 ms long with 16 interleaves required to fill kx-ky space. All 8 kz for a given spiral interleaf were acquired in a single cardiac cycle (AW = 88 ms). Acquisition duration was 34 cardiac cycles assuming 100% respiratory efficiency (alternate R-wave gating). 3D imaging was performed after the acquisition of a stack of conventional clinical breath-hold 2D LGE images in 10 patients (acquired resolution: 1.4 × 1.8 mm reconstructed to 0.7 × 0.7 mm). The 2D images had slice thickness of 7 mm with slice gap of 3 mm. Acquisition duration was 14 cardiac cycles per slice (alternate R-wave gating). LV blood SNR and blood-myocardium CNR in conventional 2D and spiral 3D scans were compared in 3 matched slices (basal, mid and apical) using paired Wilcoxon signed rank test.

## Results

**Figure 1:**
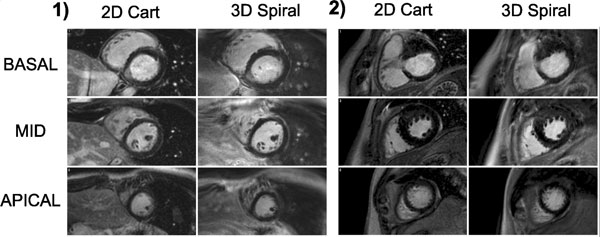
**Example LGE images from two patients showing the three LV short axis slices (Basal +30 mm, Mid 0 mm, Apical -30 mm) used for SNR and CNR measurements**. The left column shows standard 2D Cartesian images and the right column shows matched slices from the 3D spiral acquisitions. In both patients, image quality of 2D Cartesian and 3D spiral acquisitions are comparable over the LV. ROI for SNR and CNR measurements were drawn on the relevant 2D slices and transferred to the respective 3D spiral images.

**Figure 2:**
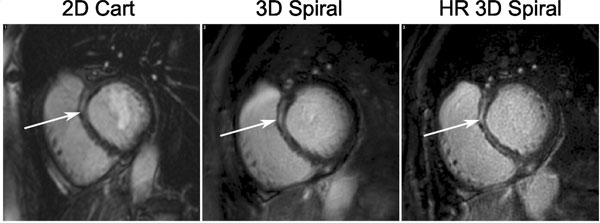
**Images of the Basal slice from a third patient showing enhancement in the septum (arrowed)**. From left to right the images are from the 2D Cartesian, 3D spiral and a higher through plane resolution (HR - 16 × 1.5 mm slices) scans. The higher resolution scan shows better definition of the enhanced region in the mid wall.

SNR measurements were significantly higher and CNR were borderline significantly higher for the 3D spiral compared to the 2D scans (SNR: 10.96 ± 2.71 vs 13.59 ± 4.70 (p < 0.01); CNR: 9.23 ± 2.60 vs 10.52 ± 4.00 (p = 0.06)). Figure 1 shows images from 2 example patients. Image quality over the LV is similar for 2D Cartesian and 3D spiral acquisitions although some off-resonance blurring can sometimes be seen in the spiral acquisitions, despite the short spiral readout durations (e.g. inferolateral wall of the RV in patient 1). This may be improved using spatially varying off-resonance correction. Figure 2 shows basal slices from a patient with enhancement in the septum which is more defined on the 3D spiral image due to better through-plane resolution and a reduced AW. A higher through-plane resolution 3D spiral image is also shown for comparison. Spiral 3D scans had mean acquisition duration 83 s (51-144 s), allowing for multiple repetitions or high resolution examination of regions of enhancement in similar duration as the 2D stack with 8 slices taking ~240 s; shorter AWs may also have reduced motion blurring.

## Conclusions

Navigator-gated 3D spiral LGE imaging allows contiguous coverage of the LV with higher spatial resolution, shorter acquisition windows and reduced acquisition durations when compared to a stack of conventional breath-hold 2D LGE Cartesian acquisitions.

## Funding

Wellcome Trust Grant WT093953MA NIHR - National Institute for Health Research.

